# Genetic Diversity and Novel Lineages of *Anaplasma*, *Ehrlichia*, and *Coxiella*-like Endosymbionts in Ticks from a Forest Ecosystem in Northeastern China

**DOI:** 10.3390/pathogens15030301

**Published:** 2026-03-10

**Authors:** Qingzhu Huang, Zhongqiu Teng, Miao Lu, Yuqing Cheng, Xincheng Qin, Lupeng Dai, Junrong Liang, Tian Qin, Jianguo Xu

**Affiliations:** 1 School of Public Health, Nanjing Medical University, Nanjing 211166, China; huangqingzhu@stu.njmu.edu.cn (Q.H.); dailupeng1995@163.com (L.D.); 2 National Key Laboratory of Intelligent Tracking and Forecasting for Infectious Diseases, National Institute for Communicable Disease Control and Prevention, Chinese Center for Disease Control and Prevention, Beijing 102206, China; tengzhongqiu@icdc.cn (Z.T.); lumiao@icdc.cn (M.L.); cyuqing0723@163.com (Y.C.); qinxincheng@icdc.cn (X.Q.); liangjunrong@icdc.cn (J.L.)

**Keywords:** ticks, *Haemaphysalis japonica*, *Haemaphysalis concinna*, *Ixodes persulcatus*, *Anaplasma*, *Ehrlichia*, *Coxiella*-like endosymbionts, molecular epidemiology

## Abstract

Ticks are important vectors of bacterial pathogens with veterinary and public health significance. However, information on the diversity of tick-associated bacteria in forest ecosystems of northeastern China remains limited. In this study, 821 questing ticks were collected from Huoshankou National Forest Park in Mudanjiang City, Heilongjiang Province, and identified as *Haemaphysalis japonica*, *Hae. concinna*, and *Ixodes persulcatus*. Molecular screening based on *rrs* gene amplification detected *Anaplasma*, *Ehrlichia*, and *Coxiella*-like endosymbionts (CLE), which were further characterized using multilocus phylogenetic analyses. *Anaplasma bovis* was detected in *Hae. concinna* and exhibited two distinct genotypes. In addition, a potentially novel *Anaplasma* lineage was identified in *I. persulcatus*. Phylogenetic analyses based on the *rrs*, *gltA*, and *groEL* genes consistently placed this lineage in a separate clade from currently recognized *Anaplasma* species. Furthermore, two genetically distinct *Ehrlichia* lineages were detected in *Hae. japonica* and *Hae. concinna*. Two lineages of tick-associated CLE were also characterized using five genetic genes. Overall, these findings demonstrate considerable genetic diversity of tick-associated bacteria in forest ecosystems of northeastern China and expand current understanding of their molecular diversity. Further studies incorporating broader sampling and genome-based analyses will be necessary to clarify their taxonomic status and epidemiological relevance.

## 1. Introduction

Ticks are among the most important hematophagous arthropods worldwide and serve as efficient vectors for a wide range of bacterial pathogens affecting both human and animal health [1,2]. Tick-borne bacterial pathogens mainly include members of the genera *Anaplasma*, *Ehrlichia*, *Rickettsia*, *Coxiella*, and *Borrelia*, many of which are responsible for emerging or re-emerging vector-borne diseases globally [3]. Due to their wide geographical distribution and complex enzootic transmission cycles involving wildlife, livestock, and humans, tick-borne diseases represent a persistent public health concern, particularly in regions with abundant forest ecosystems and wildlife hosts [3,4,5,6].

Members of the family Anaplasmataceae, including the genera *Anaplasma* and *Ehrlichia*, are Gram-negative obligate intracellular bacteria that are primarily maintained in nature through transmission cycles between ixodid ticks and vertebrate hosts [7,8]. Several *Anaplasma* species are of veterinary and medical importance, causing economically significant diseases in livestock as well as zoonotic infections in humans [9,10,11]. Among them, *Anaplasma bovis* is a monocytotropic species that has been widely detected in cattle and other ruminants in Africa, Asia, and South America, where infection is often associated with fever, anemia, reduced productivity, and occasional mortality [12,13]. Although *A. bovis* infection in animals is frequently asymptomatic, increasing evidence suggests that this pathogen also possesses zoonotic potential, as sporadic human infections have been reported in recent years [14,15,16,17].

The genus *Ehrlichia*, belongs to the family Anaplasmataceae and currently comprises several recognized species that can cause ehrlichiosis in humans and animals [18,19,20]. Previous studies have demonstrated that *Ehrlichia* species are widely distributed in ticks across Europe, Asia, and North America, and multiple unclassified *Ehrlichia* variants have also been detected in natural tick populations [21]. In Asia, a number of *Ehrlichia*-like bacteria have been identified in *Ixodes* and *Haemaphysalis* ticks, highlighting the substantial but incompletely characterized diversity of this genus in the region [8,22,23,24,25,26,27,28,29].

In addition to Anaplasmataceae, the genus *Coxiella* (family Coxiellaceae) includes both pathogenic species, such as *Coxiella burnetii*, the causative agent of Q fever, and a large group of *Coxiella*-like endosymbionts (CLE) that are commonly associated with ticks [30,31,32]. Although CLE are generally regarded as non-pathogenic to vertebrate hosts, previous studies have indicated that they are widespread among *Ixodes* ticks and exhibit substantial genetic diversity [33]. However, information on the diversity and distribution of CLE in natural tick populations remains limited, particularly in forest ecosystems where ticks and wildlife hosts are abundant [34].

Northeastern Asia, characterized by extensive forest coverage and rich wildlife resources, provides suitable ecological conditions for ticks and tick-borne pathogens [21]. However, compared with other regions of China, systematic molecular investigations of *Anaplasma*, *Ehrlichia*, and *Coxiella* in ticks from northeastern forest ecosystems remain limited, and the genetic diversity of these bacteria in this region is still poorly understood [34,35,36]. Therefore, the aim of this study was to investigate the presence and genetic diversity of *Anaplasma*, *Ehrlichia*, and *Coxiella*-like endosymbionts in ticks collected from a forest ecosystem in northeastern China using molecular detection and phylogenetic analyses.

## 2. Materials and Methods

### 2.1. Sample Collection and Processing

**Figure 1:**
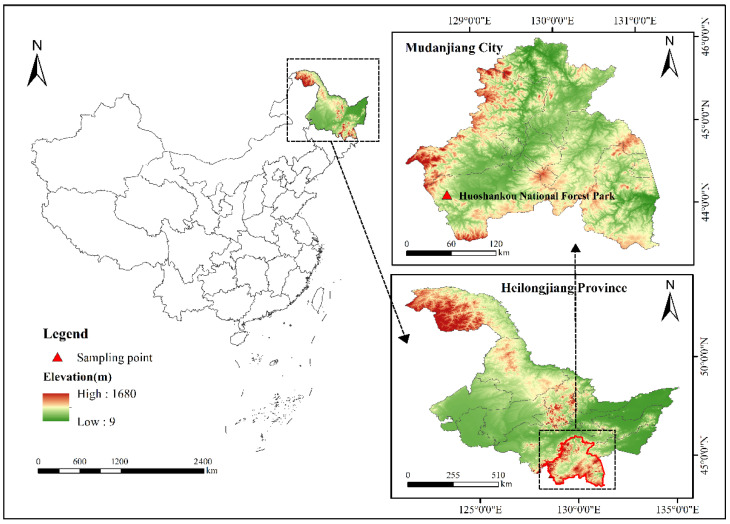
Sampling sites in Mudanjiang, Heilongjiang province.

From April to May 2025, tick collection was conducted using the standard flagging method in Huoshankou National Forest Park, Mudanjiang City, Heilongjiang Province (Figure 1) [37]. Collected ticks were identified morphologically under an optical microscope using a taxonomic key [38]. After collection, ticks were maintained in an artificial climate incubator (25 °C, relative humidity 95%, 12 h light/12 h dark) until nucleic acid extraction.

Sampling sites are marked with red triangles. The map was constructed using ArcGIS v10.8.2 software. The basemap shapefiles were downloaded from the Chinese Resource and Environmental Science Data Platform (http://www.resdc.cn/DOI:10.12078/2023010102, accessed on 15 October 2025).

To reduce external contamination, each tick was rinsed three times with 75% ethanol followed by phosphate-buffered saline (PBS), air-dried, and then 0.3 mL of PBS was added. Individual homogenization was performed using a Mixer MM 400 (Retsch, Haan, Germany). Total genomic DNA was extracted using the DNeasy Blood & Tissue Kit (Qiagen, Hilden, Germany) according to the manufacturer’s instructions, eluted with 100 µL AE buffer, and stored at 4 °C. A negative control (nuclease-free water instead of sample) was set up for each batch of extraction. DNA extraction, PCR amplification, and product analysis were performed in physically partitioned laboratories to avoid cross-contamination.

### 2.2. PCR Detection and Amplification of Key Genes

To confirm tick species classification, some ticks were randomly selected, and the mitochondrial cytochrome oxidase I (*COI*) gene was sequenced and analyzed using the LCO1490 and HCO2198 primer pairs [39].

Nested PCR was used to amplify the conserved region of the 16S rRNA (*rrs*) gene for molecular screening of *Anaplasma*, *Ehrlichia*, and *Coxiella*-like endosymbionts in tick samples [40,41]. All PCR reactions included a negative control (nuclease-free water as template) and a positive control (DNA from a known positive sample). To further characterize positive samples, partial citrate synthase (*gltA*), 60 kDa heat shock protein (*groEL*), and a longer fragment of the *rrs* gene were amplified from representative *Anaplasma* and *Ehrlichia*-positive samples [42]. For *Coxiella*-positive samples, *groEL*, 23S rRNA, *rpoB*, and *dnaK* genes were amplified [41]. PCR primer sequences and conditions are listed in Table S1. PCR amplification was performed on a SensoQuest LabCycler Standard P PCR system (Göttingen, Germany). After confirmation of amplified products by 1.0% agarose gel electrophoresis, bidirectional Sanger sequencing was conducted by Sangon Biotech (Shanghai) Co., Ltd. (Shanghai, China).

### 2.3. Genetic and Phylogenetic Analysis

Sequencing results were assembled and proofread using the SeqMan program in the Lasergene software package (DNASTAR Lasergene 17) to obtain high-quality consensus sequences. Homologous sequences were identified using the BLASTn algorithm implemented at the National Center for Biotechnology Information (NCBI) (https://blast.ncbi.nlm.nih.gov/Blast.cgi; accessed on 15 August 2025) [43]. The most closely related sequences and their alignment metrics are summarized in Supplementary Table S2. Individual gene sequence and concatenated sequences were used for phylogenetic analysis. The assembled gene sequences were concatenated in the order of *rrs*, *groEL*, and *gltA* for *Anaplasma* and *Ehrlichia*, and *rrs*, 23S rRNA, *dnaK*, *groEL*, and *rpoB* for *Coxiella*. Additionally, reference sequences of different genes from various strains were obtained from GenBank, from which the amplified regions were extracted and concatenated in order (Table S3). Multiple sequence alignment was performed using Clustal W in MEGA 11.0, and phylogenetic trees were constructed using the maximum likelihood (ML) method under the best-fit substitution model, with branch support evaluated through 1000 bootstrap replicates. Representative sequences were submitted to the GenBank database (accession numbers: PX961123–PX961177, PX971029–PX971115, PX978904–PX979118).

### 2.4. Statistical Analysis

The pathogen detection rate for each tick species was calculated as the number of positive samples divided by the total number of tested samples, and the 95% confidence interval was estimated using the Clopper–Pearson method. Comparisons of detection rates between different tick species were performed using the Chi-square test or Fisher’s exact test (when the expected frequency < 5), with *p* < 0.05 considered statistically significant. All statistical analyses were completed in IBM SPSS Statistics 25.0.

## 3. Results

### 3.1. Sample Collection

**Figure 2:**
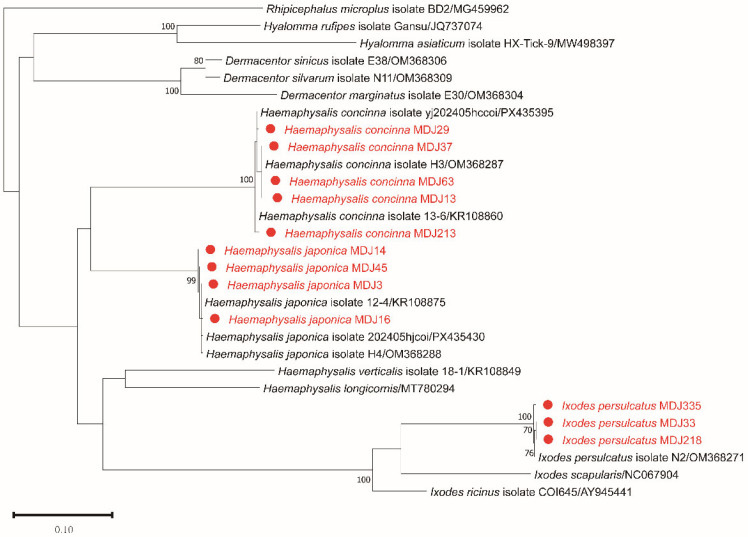
Phylogenetic analysis of tick species based on *COI* gene sequences.

A total of 821 host-seeking ticks (all adults) were collected from vegetation in Huoshankou National Forest Park, Mudanjiang. Based on morphological characteristics, these ticks belonged to three species of two genera: *Haemaphysalis japonica* (*n* = 656), *Hae. concinna* (*n* = 118), and *I. persulcatus* (*n* = 47). Further verification of the morphological identification results was conducted through *COI* gene sequence analysis. The nucleotide sequences of the amplified PCR were >99% identical with the reported *COI* reference sequences of the above three tick species. Overall, *Hae. japonica* was dominant (79.9%), followed by *Hae. concinna* (14.4%) and *I. persulcatus* (5.7%). Phylogenetic analysis indicated high genetic diversity among the samples (Figure 2). The representative *COI* sequences obtained in this study have been submitted to the GenBank database (accession numbers: PX956428–PX956439).

Phylogenetic trees were constructed using the maximum likelihood (ML) method based on the GTR + G nucleotide substitution model using *COI* gene sequences (676 bp). Bootstrap values (>70%) based on 1000 replicates are shown at the nodes. 

### 3.2. Detection and Characterization of Anaplasma

Molecular screening of 821 tick samples detected two *Anaplasma* taxa: *A. bovis* and a distinct *Anaplasma* genotype (designated the MDJ strain). *A. bovis* was detected exclusively in *Hae. concinna* (*n* = 11), whereas the MDJ strain was identified in a single *I. persulcatus* specimen (Table 1).

All *rrs* sequences obtained from the *A. bovis*-positive samples were identical. These sequences exhibited high nucleotide identity (99.8–99.9%) with previously reported *A. bovis* sequences from ruminants and wildlife in China and Japan.

**Figure 3:**
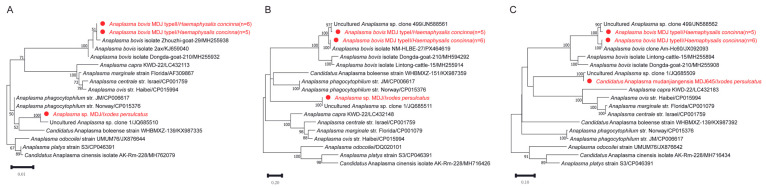
Phylogenetic tree based on the nucleotide sequences of *rrs* (**A**), *gltA* (**B**) and *groEL* (**C**) genes of *Anaplasma*.

Further characterization of the *A. bovis*-positive samples based on differences in the *gltA* and *groEL* genes revealed two distinct genotypes: Type 1 (*n* = 5) and Type 2 (*n* = 6). At the *gltA* gene, Type 1 showed approximately 96.8% nucleotide identity with uncultured *Anaplasma* sequences, whereas Type 2 exhibited higher identity values (up to 99.8%) with reported *A. bovis* sequences. Similarly, *groEL* analysis demonstrated identity values of approximately 98.3% for Type 1 and 99.7–99.8% for Type 2 compared with reference sequences. Phylogenetic analyses of *gltA* and *groEL* consistently placed both genotypes within the *A. bovis*-related clade, with Type 2 clustering with recognized *A. bovis* strains and Type 1 forming a distinct but closely related sublineage (Figure 3). To further resolve their evolutionary relationships, a concatenated phylogenetic tree was constructed based on the *rrs*, *gltA*, and *groEL* sequences. In the multilocus concatenated tree, both genotypes clustered within the *A. bovis* lineage with strong statistical support. Type 1 and Type 2 formed a distinct but closely related subclade (Figure S1).

The phylogenetic trees were inferred using the maximum likelihood (ML) method based on the nucleotide sequences of (A) *rrs* (1257 bp), (B) *gltA* (982 bp), and (C) *groEL* (1059–1289 bp). The best-fit substitution models applied were K2 + G + I for *rrs*, T92 + G + I for *gltA*, and GTR + G + I for *groEL*. Bootstrap values (>70%) based on 1000 replicates are shown at the nodes. 

In addition, a genetically distinct *Anaplasma* genotype (MDJ strain) was identified from a single *I. persulcatus* tick. The *rrs* gene sequence of the MDJ strain exhibited 99.7–100% nucleotide identity with several uncultured *Anaplasma* sequences detected in sika deer and ticks from Japan and China.

At the *gltA* and *groEL* gene, the highest nucleotide identity values were observed with uncultured *Anaplasma* sp. clone 1 from *Hae. douglasi* in Japan (99.47% for *gltA* and 99.22% for *groEL*). However, when compared with validated *Anaplasma* species, *gltA* identity values ranged from approximately 94.5% to 95.7%, and *groEL* identity values ranged from 89.2% to 93.3%, indicating marked divergence in protein-coding genes.

Multilocus phylogenetic analyses based on *rrs*, *gltA*, and *groEL* genes consistently placed the MDJ strain together with clone 1–related sequences but separate from recognized *Anaplasma* species (Figure 3). These results suggest that the MDJ strain belongs to an uncultured *Anaplasma* lineage previously reported in Japan, rather than representing a variant of any currently validated species. A concatenated phylogenetic tree based on *rrs*, *gltA*, and *groEL* sequences consistently placed the MDJ strain together with clone 1–related sequences, forming a well-supported lineage distinct from all recognized *Anaplasma* species (Figure S1). This topology was concordant with the individual gene trees.

### 3.3. Detection and Characterization of Ehrlichia

In this study, *Ehrlichia* sequences were detected in both *Hae. concinna* and *Hae. japonica*. A total of 20 positive samples were identified, including 9 from *Hae. concinna* (Type 1: *n* = 6; Type 2: *n* = 3) and 11 from *Hae. japonica* (Type 1: *n* = 8; Type 2: *n* = 3). Based on multilocus sequence comparison of *rrs*, *gltA*, and *groEL* genes, the sequences were consistently divided into two genetic types (Type 1 and Type 2).

The phylogenetic trees were inferred using the maximum likelihood (ML) method based on the nucleotide sequences of (A) *rrs* (1242 bp), (B) *gltA* (1054 bp), and (C) *groEL* (502 bp). The substitution models applied were K2 + G for *rrs*, TN92 + G + I for *gltA*, and HKY + G for *groEL*. Bootstrap values (>70%) based on 1000 replicates are shown at the nodes. 

BLASTn analysis of the *rrs* gene revealed that both types shared high nucleotide identity (99.3–100%) with uncultured *Ehrlichia* sequences previously reported from ticks in Russia and China. Specifically, Type 1 showed 99.3% sequence identity with *E. ewingii*, whereas Type 2 exhibited the highest identity (99.9%) with an *Ehrlichia* isolate detected in *Dermacentor nuttalli* from Xinjiang, China, and 99.5% identity with *E. ewingii.*

For the *gltA* gene (obtained only from Type 2), sequence identity ranged from 96.4% to 97.7% compared with related *Ehrlichia* sequences reported in China, while identity with *E. ewingii* was 89.0%. Similarly, *groEL* gene analysis showed that Type 1 shared 99.5–99.7% identity with uncultured *Ehrlichia* sequences and 95.3% identity with *E. ewingii*. Type 2 displayed 98.1–98.2% identity with related reference sequences and 94.5% identity with *E. ewingii*. Detailed BLASTn alignment metrics for all analyzed genes are provided in Supplementary Table S2.

**Figure 4:**
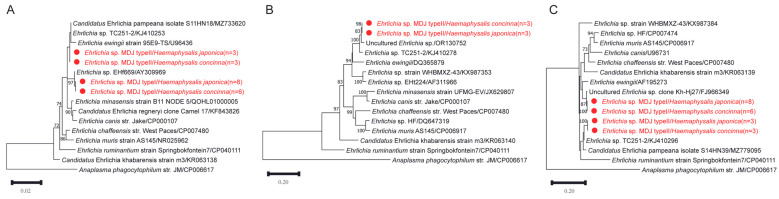
Phylogenetic tree based on the nucleotide sequences of *rrs* (**A**), *gltA* (**B**) and *groEL* (**C**) genes of *Ehrlichia*.

Although the *rrs* sequences exhibited high similarity to those of recognized *Ehrlichia* species, phylogenetic analyses revealed that both types formed distinct clades separate from recognized *Ehrlichia* species (Figure 4). This topological position remained stable in multilocus phylogenetic reconstructions, suggesting that they represent genetically distinguishable lineages within the genus *Ehrlichia* rather than confirmed novel species. Based on multilocus sequence comparisons and phylogenetic analyses, our findings confirm the presence of at least two phylogenetically distinguishable *Ehrlichia* lineages in *Hae. concinna* and *Hae. japonica*. However, additional genomic and biological characterization will be required before proposing formal taxonomic classification. In the concatenated phylogenetic tree, Type 2 formed a well-supported clade closely related to an *Ehrlichia* sp. previously identified in *D. nuttalli* from Xinjiang, China, while remaining clearly separated from all other validated *Ehrlichia* species. The overall topology was stable and consistent with the results obtained from single-gene analyses (Figure S2).

### 3.4. Genetic and Phylogenetic Analysis of Coxiella

**Figure 5:**
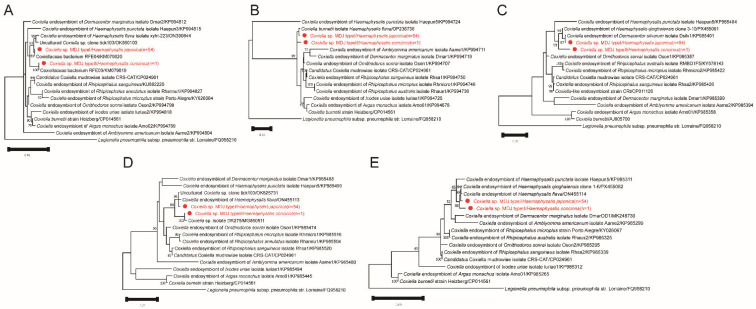
Phylogenetic tree based on the nucleotide sequences of *rrs* (**A**), 23S rRNA (**B**), *dnaK* (**C**), *groEL* (**D**), *rpoB* (**E**) genes of *Coxiella*.

Two distinct CLE sequence types were detected in tick samples in this study. *Coxiella* sp. MDJ type I was identified in 54 *Hae. japonica* ticks, whereas *Coxiella* sp. MDJ type II was detected in one *Hae. concinna* tick. For both types, five gene fragments (*rrs*, 23S rRNA, *dnaK*, *groEL*, and *rpoB*) were successfully amplified and used for multilocus sequence comparison and phylogenetic analysis (Figure 5).

The phylogenetic trees were inferred using the maximum likelihood (ML) method based on the nucleotide sequences of (A) *rrs* (1246 bp), (B) 23S rRNA (506 bp), (C) *dnaK* (562 bp), (D) *groEL* (580 bp), and (E) *rpoB* (494 bp). The substitution models applied were K2 + G for *rrs* and 23S rRNA, T92 + G for *dnaK* and *groEL*, and T92 + G + I for *rpoB*. Bootstrap values (>70%) based on 1000 replicates are shown at the nodes. 

BLASTn analysis showed that the *rrs* sequence of type I exhibited 99.9–100% nucleotide identity with previously reported tick-associated Coxiellaceae sequences from *Haemaphysalis* ticks in Russia and China. The *rrs* sequence of type II showed 99.8% identity with Coxiellaceae bacterium RFE03 from *Hae. concinna* in Russia.

At the 23S rRNA gene, type I displayed 99.0% identity with reference *Coxiella* sequences, whereas type II showed lower identity (96.8%), suggesting greater genetic divergence. For protein-coding genes, type I exhibited high nucleotide identity (approximately 96.5–99.8%) with reported tick-associated *Coxiella* endosymbionts across *dnaK*, *groEL*, and *rpoB* genes. In contrast, type II demonstrated substantially lower nucleotide identity values at several gene (approximately 90–92% for *dnaK* and *rpoB*), indicating a more distinct genetic profile. Detailed BLASTn alignment metrics for all analyzed gene are provided in Supplementary Table S2.

Phylogenetic trees reconstructed using the *rrs*, 23S rRNA, *dnaK*, *groEL*, and *rpoB* datasets consistently placed both sequence types within the tick-associated CLE clade(Figure 5). In all gene trees, type I clustered with CLE sequences previously reported from *Haemaphysalis* ticks, whereas type II formed a neighboring but distinct lineage. Bootstrap support values were high for the major nodes separating type I and type II, confirming stable genetic differentiation across gene. To further improve phylogenetic resolution, a concatenated dataset comprising *rrs*, 23S rRNA, *dnaK*, *groEL*, and *rpoB* sequences was constructed. The combined analysis consistently positioned both sequence types within the tick-associated CLE clade with strong statistical support. Type I and Type II formed two adjacent but independent lineages that clustered with previously reported CLE sequences from *Haemaphysalis* ticks. The branching pattern observed in the concatenated tree was congruent with that of the single-gene analyses, further supporting their stable genetic divergence (Figure S3).

Comprehensive multilocus sequence comparisons and phylogenetic analyses indicate that both types of sequences belong to the tick-associated CLE lineage. However, the observed inter-type differences—particularly in the protein-coding genes—suggest that Type I and Type II may represent two genetically distinct CLE lineages. Their taxonomic status still requires further confirmation through additional genomic studies.

## 4. Discussion

This study investigated tick-associated bacteria in the forest ecosystem of Huoshankou National Forest Park, Mudanjiang, in northeastern China. This region is characterized by dense vegetation and diverse wildlife hosts, providing favorable ecological conditions for tick survival and pathogen maintenance [44]. Ticks are recognized vectors of several Rickettsiales bacteria, including *Anaplasma* and *Ehrlichia* [45,46]. In this study, three tick species were identified, with *Haemaphysalis* being the dominant genus. *Hae. japonica* is one of the most common tick species in the survey. This tick is widely distributed in East Asia, including Japan, eastern China, the Korean Peninsula, and the Russian Far East [47], and is known to parasitize a variety of vertebrate hosts. However, the present study focused exclusively on questing adult ticks collected from a single site during April–May 2025, and therefore reflects bacterial diversity within this specific temporal and spatial window.

*Anaplasma bovis* is recognized as a pathogen of ruminants and has been reported in Africa and Asia [15]. In recent years, sporadic human infections have also been documented [4,16,17], although the epidemiological significance of these cases remains under investigation. In this study, *A. bovis* was detected only in *Hae. concinna* with a prevalence of 9.3%. The *rrs* sequences were identical among positive samples, whereas variation was observed at the *gltA* and *groEL* gene, allowing classification into two genotypes. Both genotypes clustered within the *A. bovis* clade in phylogenetic trees. The observed genetic differences at *gltA* and *groEL* indicate intraspecific diversity within local *A. bovis* populations. Although recombination could contribute to genetic diversification in bacteria, no recombination analysis was performed in the present study; therefore, the evolutionary mechanisms underlying this variation cannot be determined. Comparable prevalence values have been reported in ticks from northeastern China [48,49], although infection rates may vary depending on ecological conditions, host availability, and sampling design [45,50,51].

In this study, we discovered a potentially novel lineage of *Anaplasma* in *I. persulcatus*. Phylogenetic analyses based on *rrs*, *gltA*, and *groEL* consistently placed this strain in a separate clade from currently validated *Anaplasma* species. Although the *rrs* sequence showed high similarity to uncultured *Anaplasma* sequences detected in Japan [52], multilocus evidence supported its phylogenetic distinctiveness. Given the conserved nature of the *rrs* gene in the genus *Anaplasma*, high sequence similarity alone is insufficient to define species boundaries. The inclusion of more variable protein-coding genes (*gltA* and *groEL*) therefore provides more robust phylogenetic resolution.

Because this lineage was detected in only one tick and no vertebrate hosts were examined, its host range, prevalence, and pathogenic potential remain unknown. Additional sampling, genomic characterization, and taxonomic evaluation will be necessary to clarify its status. At present, we conservatively describe this strain as a distinct *Anaplasma* lineage rather than formally proposing a novel species. Importantly, phylogenetic proximity to recognized pathogenic species does not in itself demonstrate zoonotic potential, and no inference regarding human pathogenicity can be made from the present data.

The genus *Ehrlichia* belongs to the family Anaplasmataceae and consists of six recognized species: *E. canis*, *E. chaffeensis*, *E. muris*, *E. ewingii*, *E. ruminantium*, and *E. minasensis* [53,54]. These species have been detected in various ticks in China, including, *Amblyomma testudinarium*, *Hae*. *yeni*, *Hae*. *longicornis*, *I. sinensis*, *D. silvarum*, *Rhipicephalus sanguineus*, and *R. microplus* [22,55,56,57].

In this study, we also detected two genetically distinct *Ehrlichia* lineages in *Hae. concinna* and *Hae*. *japonica*. Gene alignment and phylogenetic analyses based on *rrs*, *gltA*, and *groEL* consistently showed that these two types formed independent clades. Type 1 clustered with *Ehrlichia*-related sequences from *Haemaphysalis* ticks in Russia and Japan, while Type 2 was closely related to sequences from *D*. *nuttalli* in Xinjiang, China and *Hae. juxtakochi* in Uruguay. Although these sequences showed phylogenetic proximity to *E. ewingii* in the *rrs*, *gltA*, and *groEL* [58,59], they were clearly separated from validated *E. ewingii* reference strains, supporting the presence of genetically distinct *Ehrlichia* lineages in ticks from northeastern China. Given that the present study analyzed only questing ticks and did not include vertebrate hosts, no inference can be made regarding their pathogenicity or epidemiological significance. Further multilocus and genome-based investigations would be required to clarify their taxonomic status and biological relevance.

Similar to other members of the order Rickettsiales, the genus *Coxiella* (family *Coxiellaceae*) comprises obligate intracellular bacterial genus [60]. This genus includes two confirmed species (*C. burnetii* and *C. cheraxi*), one candidate species (*Candidatus* Coxiella mudrowiae), and numerous unclassified CLE [61,62]. In this study, CLE were identified in *Hae. japonica* and *Hae. concinna*. Phylogenetic analysis based on the *rrs* gene showed high similarity to previously reported *Coxiella* sequences from *Haemaphysalis* ticks in Russia. However, tree topologies inferred from additional genes (23S rRNA, *groEL*, *rpoB*, and *dnaK*) showed some differences in clustering patterns and reference affinities. This incongruence may reflect the limited availability of multilocus CLE data in public databases, where different genes are unevenly represented and not always derived from the same strains.

Importantly, we obtained sequences of five genes from the same DNA extracts, providing multilocus support for the genetic distinctiveness of these CLE lineages. Based on host association and phylogenetic placement, we provisionally designate them as *Coxiella* endosymbiont of *Hae. japonica* isolate MDJ and *Coxiella* endosymbiont of *Hae. concinna* isolate MDJ. Although multilocus evidence supports their distinctiveness, genome-level data would be required for formal species delimitation.

Ticks commonly harbor bacterial endosymbionts, including members of the genera *Coxiella*, *Rickettsia*, and *Francisella*, which are widespread components of tick microbiota [63]. These endosymbionts are thought to contribute to host nutrition, development, and reproduction. Phylogenetic analyses indicate that CLE form multiple subclusters within the broader *Coxiella* clade, highlighting their evolutionary complexity [41]. While many CLE are considered non-pathogenic, their ecological roles within tick populations warrant further investigation.

Although this study provides valuable data on the genetic diversity of tick-associated bacteria in northeastern China, several limitations should be acknowledged. Sampling was restricted to a single forest park and conducted during a limited seasonal period, which may affect representativeness. In addition, only adult questing ticks were examined, and vertebrate hosts were not included.

Furthermore, although novel *Anaplasma* and distinct *Ehrlichia* lineages were identified, conclusions regarding their prevalence, host range, or pathogenicity cannot be drawn from the present data. Future studies incorporating broader geographic sampling, vertebrate host surveillance, concatenated multilocus analyses, and genome-based approaches would provide a more comprehensive understanding of their taxonomic status and ecological significance.

## 5. Conclusions

In conclusion, this study provides molecular evidence for the genetic diversity of *Anaplasma*, *Ehrlichia*, and *Coxiella*-like endosymbionts in ticks collected from a forest ecosystem in northeastern China. *A. bovis* exhibited intraspecific genetic variation, and a putative novel *Anaplasma* lineage was identified in *I. persulcatus* based on multilocus phylogenetic analyses. In addition, two genetically distinct *Ehrlichia* lineages and two lineages of tick-associated *Coxiella*-like endosymbionts were characterized. These findings contribute to current knowledge of tick-associated bacterial diversity in northeastern China. However, further studies incorporating expanded geographic sampling, vertebrate host investigation, and genome-based analyses will be necessary to clarify their taxonomic status and epidemiological relevance.

## Figures and Tables

**Table 1:** Prevalence of *Anaplasma*, *Ehrlichia*, and *Coxiella* in ticks collected from Heilongjiang Province, China.

Tick Species	No. Tested	No. Infected Ticks (%, 95% CI)			
		*A. bovis*	*Anaplasma* sp. MDJ	*Ehrlichia* sp.	*Coxiella* sp.
*Hae. japonica*	656	0	0	11 (1.68, 0.84–2.98)	54 (8.23, 6.24–10.60)
*Hae. concinna*	118	11 (9.32, 4.75–16.07)	0	9 (7.63, 3.55–13.99)	1 (0.85, 0.02–4.63)
*I. persulcatus*	47	0	1 (2.13, 0.05–11.29)	0	0
Total	821	11 (1.34, 0.67–2.38)	1 (0.12, 0.00–0.68)	20 (2.44, 1.49–3.74)	55 (6.70, 5.13–8.63)
*p* value		<0.001 ^b^	NA ^a^	<0.001 ^b^	0.002 ^b^

Values in parentheses represent 95% confidence intervals. NA, not applicable. ^a^ Statistical comparison was not performed due to the low number and uneven distribution of positive samples among tick species. ^b^ Fisher’s exact test was used to compare infection rates among tick species.

## Data Availability

The original contributions presented in this study are included in the article/Supplementary Materials. Further inquiries can be directed to the corresponding author.

## Supplementary Materials

The following supporting information can be downloaded at https://www.mdpi.com/article/10.3390/pathogens15030301/s1, Table S1: Nucleotide sequence of primers used in the study. Table S2. Summary of BLASTn alignment metrics for representative sequences used in phylogenetic analyses. Table S3. GenBank accession numbers for validated strains used for concatenated sequence. Figure S1. Phylogenetic tree based on concatenated nucleotide sequences of *Anaplasma*. The phylogenetic tree was inferred using the maximum likelihood (ML) method based on concatenated sequences of *rrs*, *groEL*, and *gltA* genes. The substitution model applied was T92 + G + I. Bootstrap values (>70%) based on 1000 replicates are shown at the nodes. Sequences obtained in this study are indicated in red and marked with circles. Figure S2. Phylogenetic tree based on concatenated nucleotide sequences of *Ehrlichia*. The phylogenetic tree was inferred using the maximum likelihood (ML) method based on concatenated sequences of *rrs*, *groEL*, and *gltA* genes. The substitution model applied was TN93 + G. Bootstrap values (>70%) based on 1000 replicates are shown at the nodes. Sequences obtained in this study are indicated in red and marked with circles. Figure S3. Phylogenetic tree based on concatenated nucleotide sequences of *Coxiella*-like endosymbionts. The phylogenetic tree was inferred using the maximum likelihood (ML) method based on concatenated sequences of *rrs*, 23S rRNA, *dnaK*, *groEL*, and *rpoB* genes. The substitution model applied was T92 + G. Bootstrap values (>70%) based on 1000 replicates are shown at the nodes. Sequences obtained in this study are indicated in red and marked with circles.
