# Supplementary material for: Genetic Diversity and Novel Lineages of Anaplasma, Ehrlichia, and Coxiella-like Endosymbionts in Ticks from a Forest Ecosystem in Northeastern China
Source: Pathogens. 2026 Mar 10;15(3):301. doi: 10.3390/pathogens15030301 (PMC13028735; doi:10.3390/pathogens15030301)
Supplement: Supplementary file 1 [file pathogens-15-00301-s001.zip › Figure S3.pdf]

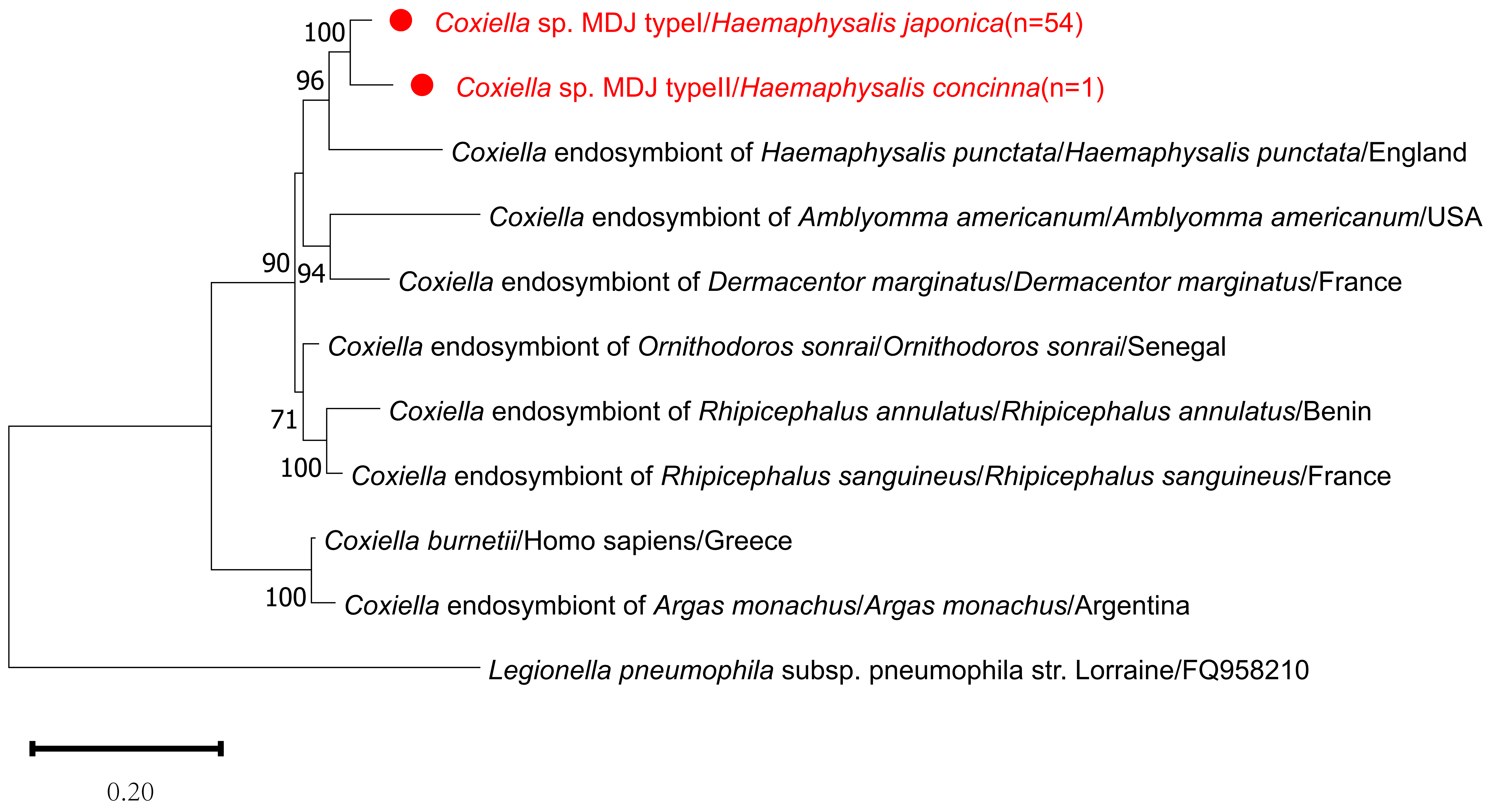

Figure S3. Phylogenetic tree based on concatenated nucleotide sequences of *Coxiella*-like endosymbionts.
