# Supplementary material for: Genetic Diversity and Novel Lineages of Anaplasma, Ehrlichia, and Coxiella-like Endosymbionts in Ticks from a Forest Ecosystem in Northeastern China
Source: Pathogens. 2026 Mar 10;15(3):301. doi: 10.3390/pathogens15030301 (PMC13028735; doi:10.3390/pathogens15030301)
Supplement: Supplementary file 1 [file pathogens-15-00301-s001.zip › Table S1.pdf]

Table S1. Nucleotide sequence of primers used in the study.

Primer	Cycle	Bacteria	Gene	Sequence	Anticipated Amplicon Length
Ana-F	1, 2	*Anaplasma*	16S	5-GATAGCCACTRGAAGTGGT-3	900 bp
Ana-R1	1	*Anaplasma*	16S	5-CGTGCTGACTTGACATCAT-3	
Ana-R2	2	*Anaplasma*	16S	5-CATCTCACGACACGAGCTG-3	
Ana1	1	*Anaplasma*	16S	5-GAACGAACGCTGGCGGCAAGC-3	500 bp
Ana2	1	*Anaplasma*	16S	5-AGTAYCGRACCAGATAGCCGC-3	
Ana3	2	*Anaplasma*	16S	5-TGCATAGGAATCTACCTAG-3	
Ana4	2	*Anaplasma*	16S	5-CTAGGAATTCCGCTATCCTCT-3	
Ab-glt-F1	1	*A. bovis*	*gltA*	5-TTYATAGATGGRGATRAGGGC-3	1000 bp
Ab-glt-F2	2	*A. bovis*	*gltA*	5-AGATGGRGATRAGGGCATYCT-3	
Ab-glt-R	1, 2	*A. bovis*	*gltA*	5-AHCATTTCATRCCAYTGRG-3	
Ab-gro-F	1, 2	*A. bovis*	*groEL*	5-ACTGCTGGACCGAAGGGCTT-3	850bp
Ab-gro-R1	1	*A. bovis*	*groEL*	5-CAAAGTGATGTCCTCCATCT-3	
Ab-gro-R2	2	*A. bovis*	*groEL*	5-GCTATGTCRCCVAGCATGTCT-3	
Ab-EL-F1	1	*A. bovis*	*groEL*	5-TTGCTAAATCTGGAAGRCCAC-3	800bp
Ab-EL-F2	2	*A. bovis*	*groEL*	5-GARGACGTTGAGGGTGAAGC-3	
Ab-EL-R	1, 2	*A. bovis*	*groEL*	5-CATAAATACYGCCGCRAGAG-3	
Ap-glt-F1	1	*A. phagocytophilum*	*gltA*	5-CAAGTGGTTGATATCACAACC-3	1020bp
Ap-glt-F2	2	*A. phagocytophilum*	*gltA*	5-TATAGGCAGAGCAATATACTC-3	
Ap-glt-R	1, 2	*A. phagocytophilum*	*gltA*	5-CAAAGCCTGCATTCTTAACTC-3	
Ap-gro-F1	1	*A. phagocytophilum*	*groEL*	5-TAGGAGGTCCAAAGATGTCAAATAC-3	1000bp
Ap-gro-F2	2	*A. phagocytophilum*	*groEL*	5-CACGGGTGAGCTGTTTAGATAAG-3	
Ap-gro-R	1, 2	*A. phagocytophilum*	*groEL*	5-CTTGACTTCAACTTCACTAGAG-3	
Ehr-F	1, 2	*Ehrlichia*	16S	5-GAATAGCCATTAGAAATGAT-3	550 bp
Ehr-R1	1	*Ehrlichia*	16S	5-GTCAGTATCGAACCAGATAG-3	
Ehr-R2	2	*Ehrlichia*	16S	5-GTATCGAACCAGATAGCCG-3	
E-rrs-F1	1	*Ehrlichia*	16S	5-CGGCTATCTGGTTCGATAC-3	750 bp
E-rrs-F2	2	*Ehrlichia*	16S	5-CTATCTGGTTCGATACTGAC-3	
E-rrs-R	1, 2	*Ehrlichia*	16S	5-GCTTCCTTKCGGTTAGCAC-3	
E-gltA-F1	1	*Ehrlichia*	*gltA*	5-CAGGMTTTATGTCTACTGCTGCTTG-3	1000bp
E-gltA-F2	2	*Ehrlichia*	*gltA*	5-ATGTCWACTGCTGCTTGTGA-3	
E-gltA-R	1, 2	*Ehrlichia*	*gltA*	5-CCAGTATATAAYTGACGWGGACG-3	
E-gro-F1	1	*Ehrlichia*	*groEL*	5-TGGGCTGGYAATGAAATTGA-3	1100bp
E-gro-F2	2	*Ehrlichia*	*groEL*	5-AACATGGCAAATGTAGTTGT-3	
E-gro-R	1, 2	*Ehrlichia*	*groEL*	5-TCAACAGCAGCTCTAGTT-3	
Cox-F1	1	*Coxiella*	16S	5-CGTAGGAATCTACCTTRTAGWGG-3	620bp
Cox-R1	1	*Coxiella*	16S	5-ACTYYCCAACAGCTAGTTCTCA-3	
Cox-F2	2	*Coxiella*	16S	5-TGAGAACTAGCTGTTGGRRAGT-3	
Cox-R2	2	*Coxiella*	16S	5-GCCTACCCGCTTCTGGTACAATT-3	
Rla16SF1	1	*Coxiella*	16S	5-CAGTAAARRTTTCGGYCTTTAYGGG-3	700bp
Rla16SR1	1	*Coxiella*	16S	5-CAAACCTAGTCAACCACCTACACG-3	
Rla16SF2	2	*Coxiella*	16S	5-CGTGTAGGTGGTTGACTAGGTTTG-3	
Rla16SR2	2	*Coxiella*	16S	5-GGATTGGCTCCCCCTCGCGGGTTGG-3	
Cox23SF1	1	*Coxiella*	23S	5-GCCTGCGAWAAGCTTCGGGGAG-3	530bp
Cox23SR1	2	*Coxiella*	23S	5-TCGYTCGGTTTCGGGTCKACTC-3	
Cox23SF2	2	*Coxiella*	23S	5-GATCCGGAGATWTCYGAATGGGG-3	
Cox23SR2	1	*Coxiella*	23S	5-CTCCTAKCCACASCTCATCCCC-3	
CoxGrF1	1	*Coxiella*	*groEL*	5-TTTGAAAAYATGGGCGCKCAAATGGT-3	620bp
CoxGrR1	2	*Coxiella*	*groEL*	5-CCAAARCCAGGTGCTTTYAC-3	
CoxGrF2	2	*Coxiella*	*groEL*	5-GAAGTGGCTTCGCRTACWTCAGACG-3	
CoxGrR2	1	*Coxiella*	*groEL*	5-CGRTCRCCAAARCCAGGTGC-3	
CoxrpoF1	1	*Coxiella*	*rpoB*	5-GGGCGNCAYGGWAAYAAAGGSGT-3	540bp
CoxrpoR1	1	*Coxiella*	*rpoB*	5-CACCRAAHCGTTGACCRCCAAATTG-3	
CoxrpoF2	2	*Coxiella*	*rpoB*	5-TCGAAGAYATGCCYTATTTAGAAG-3	
CoxrpoR2	2	*Coxiella*	*rpoB*	5-AGCTTTMCCACCSARGGGTTGCTG-3	
CoxdnaF1	1	*Coxiella*	*dnaK*	5-CGTCARGCRACGAARGATGCA-3	600bp
CoxdnaF2	2	*Coxiella*	*dnaK*	5-GAAGTGGATGGCGARCAYCAATT-3	
CoxdnaR	1, 2	*Coxiella*	*dnaK*	5-CGTCATGAYKCCGCCYAAGG-3	
