# Supplementary material for: Genetic Diversity and Novel Lineages of Anaplasma, Ehrlichia, and Coxiella-like Endosymbionts in Ticks from a Forest Ecosystem in Northeastern China
Source: Pathogens. 2026 Mar 10;15(3):301. doi: 10.3390/pathogens15030301 (PMC13028735; doi:10.3390/pathogens15030301)
Supplement: Supplementary file 1 [file pathogens-15-00301-s001.zip › Table S2.pdf]

Table S2. Summary of BLASTn alignment metrics for representative sequences used in phylogenetic analyses.

Query ID	Gene	Closest BLAST Hit (Description)	Accession No.	Host	Country	Query Cover (%)	Percent Identity (%)	E-Value
A. bovis typeI&typeII	*rrs*	Uncultured *Anaplasma* sp. clone sh65-4	KM186933	*Procapra gutturosa*	China	100	99.92	0.0
		Uncultured *Anaplasma* sp. clone sh65-5	KM186934	*Procapra gutturosa*	China	100	99.92	0.0
		A. bovis isolate Zhouzhi-goat-29	MH255938	goat	China	100	99.84	0.0
		A. bovis strain raccoon109	GU937011	raccoon	Japan	100	99.84	0.0
A. bovis typeI	*gltA*	Uncultured *Anaplasma* sp. clone 499	JN588561	raccoon	Japan	99	96.82	0.0
		Anaplasma bovis isolate NM-HLBE-27	PX464619	*Hae. concinna*	China	92	93.60	0.0
A. bovis typeII	*gltA*	Uncultured *Anaplasma* sp. clone 499	JN588561	*raccoon*	Japan	99	94.16	0.0
		Anaplasma bovis isolate NM-HLBE-27	PX464619	*Hae. concinna*	China	92	99.77	0.0
A. bovis typeI	*groEL*	Uncultured *Anaplasma* sp. clone 499	JN588562	*raccoon*	Japan	95	98.32	0.0
A. bovis typeII	*groEL*	*A. bovis* clone Am-Hc60	JX092093	*Hae. concinna*	Russia	100	99.84	0.0
		A. bovis clone Kh-Hc215	JX092095	*Hae. concinna*	Russia	100	99.76	0.0
		Uncultured *Anaplasma* sp. clone 499	JN588562	raccoon	Japan	95	96.64	0.0
Anaplasma MDJ strain	*rrs*	Uncultured *Anaplasma* sp. clone 1	JN055357	sika deer	Japan	100	100	0.0
		Anaplasma sp. Ac52D	AB588974	sika deer	Japan	100	100	0.0
		Anaplasma sp. Ac30B	AB588976	sika deer	Japan	100	100	0.0
		Uncultured *Anaplasma* sp. clone 1	JQ685510	*Hae. douglasi*	Japan	52	99.67	0.0
		Anaplasma phagocytophilum str. Norway	CP046639	Sheep	Norway	100	98.88	0.0
Anaplasma MDJ strain	*gltA*	Uncultured *Anaplasma* sp. clone 1	JQ685511	*Hae. douglasi*	Japan	86	99.47	0.0
		Uncultured *Anaplasma* sp. clone APJ-H2B	JX474557	*Hae. megaspinosa*	Japan	86	94.50	0.0
		Uncultured *Anaplasma* sp. clone SD-3	JQ701724	sika deer	Japan	86	95.67	0.0
		Uncultured *Anaplasma* sp. clone APJ18A	JX474550	horse	Japan	86	95.15	0.0
		Anaplasma phagocytophilum strain ZJ54	KP076357	goat	China	42	92.37	7e-143
Anaplasma MDJ strain	*groEL*	Uncultured *Anaplasma* sp. clone 1	JQ685509	*Hae. douglasi*	Japan	100	99.92	0.0
		Uncultured *Anaplasma* sp. clone Oar40	MN431825	*Ovis aries*	Tunisia	79	93.27	0.0
		Uncultured *Anaplasma* sp. clone Dongda-goat-124	MG869410	goat	China	100	89.21	0.0
		Anaplasma phagocytophilum isolate Omsk-698_Ip	MN701642	*I. persulcatus*	Russia	99	79.12	0.0
Ehrlichia sp. typeI	*rrs*	Uncultured *Ehrlichia* sp. clone Kh-Hj27	FJ966350	*Hae. japonica*	Russia	96	100	0.0
		Uncultured *Ehrlichia* sp. clone NMXAM123	PX452794	*I. persulcatus*	China	100	99.68	0.0
		Uncultured *Ehrlichia* sp. clone Dehong-18	OL838198	*R. microplus*	China	100	99.35	0.0
		Uncultured *Ehrlichia* sp. clone Liuzhi89	OP047994	*R. microplus*	China	100	99.35	0.0
		Ehrlichia sp. TC251-2	KJ410253	*D. nuttalli*	China	100	99.35	0.0
		Ehrlichia ewingii strain 95E9-TS	U96436	Dog	USA	100	99.27	0.0
		Ehrlichia chaffeensis strain Shawan pet dog	MZ433240	Dog	China	100	98.85	0.0
Ehrlichia sp. typeII	*rrs*	*Ehrlichia* sp. TC251-2	KJ410253	*D. nuttalli*	China	100	99.92	0.0
		Ehrlichia ewingii strain 95E9-TS	U96436	Dog	USA	100	99.51	0.0
		Ehrlichia chaffeensis strain Shawan pet dog	MZ433240	Dog	China	100	99.35	0.0
Ehrlichia sp. typeII	*gltA*	*Ehrlichia* sp. TC249-2	KJ410276	*D. nuttalli*	China	80	96.80	0.0
		Ehrlichia sp. TC251-2	KJ410278	*D. nuttalli*	China	82	96.76	0.0
		Ehrlichia sp. TC248-16	KJ410275	*D. nuttalli*	China	98	96.62	0.0

		Ehrlichia ewingii	DQ365879	*Amblyomma americanum*	Panola Mountain	96	89.02	0.0
		Ehrlichia chaffeensis str. Saint Vincent	CP007478	*Homo sapiens*	USA	100	84.27	0.0
Ehrlichia sp. typeI	*groEL*	Uncultured *Ehrlichia* sp. clone Kh-Hj27	FJ966349	*Hae. japonica*	Russia	100	99.59	0.0
		Ehrlichia sp. TC249-2	KJ410295	*D. nuttalli*	China	100	94.90	0.0
		Ehrlichia sp. TC251-2	KJ410296	*D. nuttalli*	China	100	94.90	0.0
		Ehrlichia sp. TC248-16	KJ410294	*D. nuttalli*	China	100	94.90	0.0
		Ehrlichia ewingii	AF195273	*Homo sapiens*	USA	100	95.29	0.0
		Ehrlichia chaffeensis str. Saint Vincent	CP007478	*Homo sapiens*	USA	100	92.62	0.0
Ehrlichia sp. typeII	*groEL*	*Ehrlichia* sp. TC249-2	KJ410295	*D. nuttalli*	China	100	98.16	0.0
		Ehrlichia sp. TC251-2	KJ410296	*D. nuttalli*	China	100	98.16	0.0
		Ehrlichia sp. TC248-16	KJ410294	*D. nuttalli*	China	100	98.16	0.0
		Ehrlichia ewingii	AF195273	*Homo sapiens*	USA	100	94.47	0.0
		Ehrlichia chaffeensis str. Saint Vincent	CP007478	*Homo sapiens*	USA	100	91.60	0.0
Coxiella sp. typeI	*rrs*	*Coxiella*ceae bacterium RFE04	KM079620	*Hae. japonica*	Russia	100	100	0.0
		*Coxiella*ceae bacterium RFE05	KM079621	*Hae. japonica*	Russia	100	99.92	0.0
Coxiella sp. typeII	*rrs*	*Coxiella*ceae bacterium RFE03	KM079619	*Hae. concinna*	Russia	100	99.84	0.0
		*Coxiella*ceae bacterium RFE04	KM079620	*Hae. japonica*	Russia	100	97.98	0.0
Coxiella sp. typeI	23S rRNA	*Coxiella* burnetii isolate xyht-233	OP236726	*Hae. flava*	China	100	99.00	0.0
		Coxiella burnetii isolate *Haemaphysalis flava*	OP236736	*Hae. flava*	China	100	99.00	0.0
Coxiella sp. typeII	23S rRNA	*Coxiella* burnetii isolate xyht-233	OP236726	*Hae. flava*	China	100	96.79	0.0
		Coxiella burnetii isolate *Haemaphysalis flava*	OP236736	*Hae. flava*	China	100	96.79	0.0
Coxiella sp. typeI	*dnaK*	*Coxiella* endosymbiont of *Dermacentor silvarum* isolate Dsilv1	KP985401	*D. silvarum*	China	100	99.82	0.0
		Coxiella endosymbiont of *Haemaphysalis qinghaiensis* clone 9-1	PX455093	*Hae. qinghaiensis*	China	97	97.79	0.0
		Coxiella endosymbiont of *Haemaphysalis qinghaiensis* clone 10-7	PX455094	*Hae. qinghaiensis*	China	97	97.79	0.0
		Coxiella endosymbiont of *Haemaphysalis qinghaiensis* clone 3-10	PX455091	*Hae. qinghaiensis*	China	97	97.79	0.0
		Coxiella endosymbiont of *Haemaphysalis qinghaiensis* clone 8-3	PX455092	*Hae. qinghaiensis*	China	97	97.79	0.0
		Coxiella endosymbiont of *Haemaphysalis qinghaiensis* clone 1-6	PX455089	*Hae. qinghaiensis*	China	97	97.79	0.0
		Coxiella endosymbiont of *Haemaphysalis qinghaiensis* clone 4-8	PX455090	*Hae. qinghaiensis*	China	97	97.79	0.0
		Coxiella endosymbiont of *Haemaphysalis qinghaiensis* clone 11-5	PX455095	*Hae. qinghaiensis*	China	97	97.79	0.0
		Coxiella endosymbiont of *Haemaphysalis flava*	ON455112	*Hae. flava*	China	87	96.52	0.0
Coxiella sp. typeII	*dnaK*	*Coxiella* endosymbiont of *Dermacentor silvarum* isolate Dsilv1	KP985401	*D. silvarum*	China	100	90	0.0
		Coxiella endosymbiont of *Haemaphysalis qinghaiensis* clone 9-1	PX455093	*Hae. qinghaiensis*	China	97	90.61	0.0
		Coxiella endosymbiont of *Haemaphysalis qinghaiensis* clone 10-7	PX455094	*Hae. qinghaiensis*	China	97	90.61	0.0
		Coxiella endosymbiont of *Haemaphysalis qinghaiensis* clone 3-10	PX455091	*Hae. qinghaiensis*	China	97	90.61	0.0
		Coxiella endosymbiont of *Haemaphysalis qinghaiensis* clone 8-3	PX455092	*Hae. qinghaiensis*	China	97	90.61	0.0
		Coxiella endosymbiont of *Haemaphysalis qinghaiensis* clone 1-6	PX455089	*Hae. qinghaiensis*	China	97	90.61	0.0
		Coxiella endosymbiont of *Haemaphysalis qinghaiensis* clone 4-8	PX455090	*Hae. qinghaiensis*	China	97	90.61	0.0
		Coxiella endosymbiont of *Haemaphysalis qinghaiensis* clone 11-5	PX455095	*Hae. qinghaiensis*	China	97	90.61	0.0
		Coxiella endosymbiont of *Haemaphysalis flava*	ON455112	*Hae. flava*	China	87	90.57	0.0
Coxiella sp. typeI	*groEL*	*Coxiella* endosymbiont of *Haemaphysalis flava*	ON455113	*Hae. flava*	China	100	97.58	0.0
		Uncultured *Coxiella* sp. clone tick103	OK625731	tick	China	94	98.53	0.0
		Uncultured *Coxiella* sp. clone tick106	OK625732	tick	China	94	98.53	0.0
Coxiella sp. typeII	*groEL*	*Coxiella* sp. isolate DR275	MG860511	*D. reticulatus*	Slovakia	97	98.75	0.0
		Coxiella endosymbiont of *Haemaphysalis flava*	ON455113	*Hae. flava*	China	100	91.35	0.0

		Uncultured *Coxiella* sp. clone tick103	OK625731	tick	China	94	92.29	0.0
		Uncultured *Coxiella* sp. clone tick106	OK625732	tick	China	94	92.29	0.0
Coxiella sp. typeI	*rpoB*	*Coxiella* endosymbiont of *Haemaphysalis flava*	ON455114	*Hae. flava*	China	96	97.03	0.0
		Coxiella endosymbiont of *Haemaphysalis qinghaiensis* clone 9-1	PX455086	*Hae. qinghaiensis*	China	96	96.81	0.0
		Coxiella endosymbiont of *Haemaphysalis qinghaiensis* clone 1-6	PX455082	*Hae. qinghaiensis*	China	96	96.81	0.0
		Coxiella endosymbiont of *Haemaphysalis qinghaiensis* clone 4-8	PX455084	*Hae. qinghaiensis*	China	96	96.81	0.0
		Coxiella endosymbiont of *Haemaphysalis qinghaiensis* clone 3-10	PX455083	*Hae. qinghaiensis*	China	96	96.60	0.0
		Coxiella endosymbiont of *Haemaphysalis qinghaiensis* clone 11-5	PX455088	*Hae. qinghaiensis*	China	96	96.60	0.0
		Coxiella endosymbiont of *Haemaphysalis qinghaiensis* clone 8-3	PX455085	*Hae. qinghaiensis*	China	96	96.60	0.0
		Coxiella endosymbiont of *Haemaphysalis qinghaiensis* clone 10-7	PX455087	*Hae. qinghaiensis*	China	96	96.60	0.0
Coxiella sp. typeII	*rpoB*	*Coxiella* endosymbiont of *Haemaphysalis flava*	ON455114	*Hae. flava*	China	96	91.51	0.0
		Coxiella endosymbiont of *Haemaphysalis qinghaiensis* clone 9-1	PX455086	*Hae. qinghaiensis*	China	96	92.13	0.0
		Coxiella endosymbiont of *Haemaphysalis qinghaiensis* clone 1-6	PX455082	*Hae. qinghaiensis*	China	96	92.13	0.0
		Coxiella endosymbiont of *Haemaphysalis qinghaiensis* clone 4-8	PX455084	*Hae. qinghaiensis*	China	96	92.13	0.0
		Coxiella endosymbiont of *Haemaphysalis qinghaiensis* clone 3-10	PX455083	*Hae. qinghaiensis*	China	96	91.91	0.0
		Coxiella endosymbiont of *Haemaphysalis qinghaiensis* clone 11-5	PX455088	*Hae. qinghaiensis*	China	96	91.91	0.0
		Coxiella endosymbiont of *Haemaphysalis qinghaiensis* clone 8-3	PX455085	*Hae. qinghaiensis*	China	96	91.91	0.0
		Coxiella endosymbiont of *Haemaphysalis qinghaiensis* clone 10-7	PX455087	*Hae. qinghaiensis*	China	96	91.91	0.0
