# Supplementary material for: Genetic Diversity and Novel Lineages of Anaplasma, Ehrlichia, and Coxiella-like Endosymbionts in Ticks from a Forest Ecosystem in Northeastern China
Source: Pathogens. 2026 Mar 10;15(3):301. doi: 10.3390/pathogens15030301 (PMC13028735; doi:10.3390/pathogens15030301)
Supplement: Supplementary file 1 [file pathogens-15-00301-s001.zip › Table S3.pdf]

Table S3. GenBank accession numbers for validated strains used for concatenated sequence.

Species	Strain	Genome (Accession no.)	GenBank Accession No.					
			rrs	*gltA*	*groEL*	23S rRNA	*dnaK*	*rpoB*
A. platys	S3	CP046391	†	†	†	NA	NA	NA
A. bovis	Dongda-goat-210	‡	MH255932	MH594292	MH255908	NA	NA	NA
A. capra	KWD-22	‡	LC432113	LC432148	LC432183	NA	NA	NA
A. centrale	Israel	CP001759	†	†	†	NA	NA	NA
A. marginale	Florida	CP001079	†	†	†	NA	NA	NA
A. ovis	Haibei	CP015994	†	†	†	NA	NA	NA
Un. A. sp.	*clone 1*	‡	JQ685510	JQ685511	JQ685509	NA	NA	NA
A. phagocytophilum	JM	CP006617	†	†	†	NA	NA	NA
A. phagocytophilum	Norway	CP015376	†	†	†	NA	NA	NA
Ca. A. boleense	WHBMXZ-139	‡	KX987335	KX987361	KX987392	NA	NA	NA
Ca. A. cinensis	AK-Rm-228	‡	MH762079	MH716426	MH716434	NA	NA	NA
Ca. E. khabarensis	m3	‡	KR063138	KR063140	KR063139	NA	NA	NA
E. canis	Jake	CP000107	†	†	†	NA	NA	NA
E. chaffeensis	West Paces	CP007480	†	†	†	NA	NA	NA
E. minasensis	UFMG-EV	‡	NR1488001	JX629807	JX629806	NA	NA	NA
E. muris	AS145	CP006917	†	†	†	NA	NA	NA
E. ruminantium	Springbokfontein7	CP040111	†	†	†	NA	NA	NA
E. sp.	TC251-2	‡	KJ410253	KJ410278	KJ410296	NA	NA	NA
C. burnetii	Heizberg	CP014561	†	NA	†	†	†	†
C. endosymbiont of Amblyomma americanum	Aame2	‡	KP994804	NA	KP985481	KP994712	KP985394	KP985299
C. endosymbiont of Argas monachus	Amo02	‡	KP994769	NA	KP985446	KP994679	KP985359	KP985266
C. endosymbiont of Dermacentor marginatus	Dmar2	‡	KP994812	NA	KP985489	KP994720	KP985400	KP985307
C. endosymbiont of Haemaphysalis punctata	Haepun3	‡	KP994815	NA	KP985492	KP994723	KP985403	KP985310
C. endosymbiont of Ornithodoros sonrai	Oson2	‡	KP994798	NA	KP985475	KP994708	KP985388	KP985295
C. endosymbiont of Rhipicephalus annulatus	Rhannu1	‡	KP994827	NA	KP985504	KP994734	KP985409	KP985322
C. endosymbiont of Rhipicephalus sanguineus	Rhsa1	‡	KP994843	NA	KP985520	KP994750	KP985425	KP985338
Legionella pneumophila subsp. pneumophila	Lorraine	FQ958210	†	NA	†	†	†	†

† = Complete genome available in GenBank; ‡ = Individual genes sequences available in GenBank; NA = Gene sequences not available in GenBank.
