# Supplementary figures and images for: Genetic Diversity and Novel Lineages of Anaplasma, Ehrlichia, and Coxiella-like Endosymbionts in Ticks from a Forest Ecosystem in Northeastern China
Source: Pathogens. 2026 Mar 10;15(3):301. doi: 10.3390/pathogens15030301 (PMC13028735; doi:10.3390/pathogens15030301)

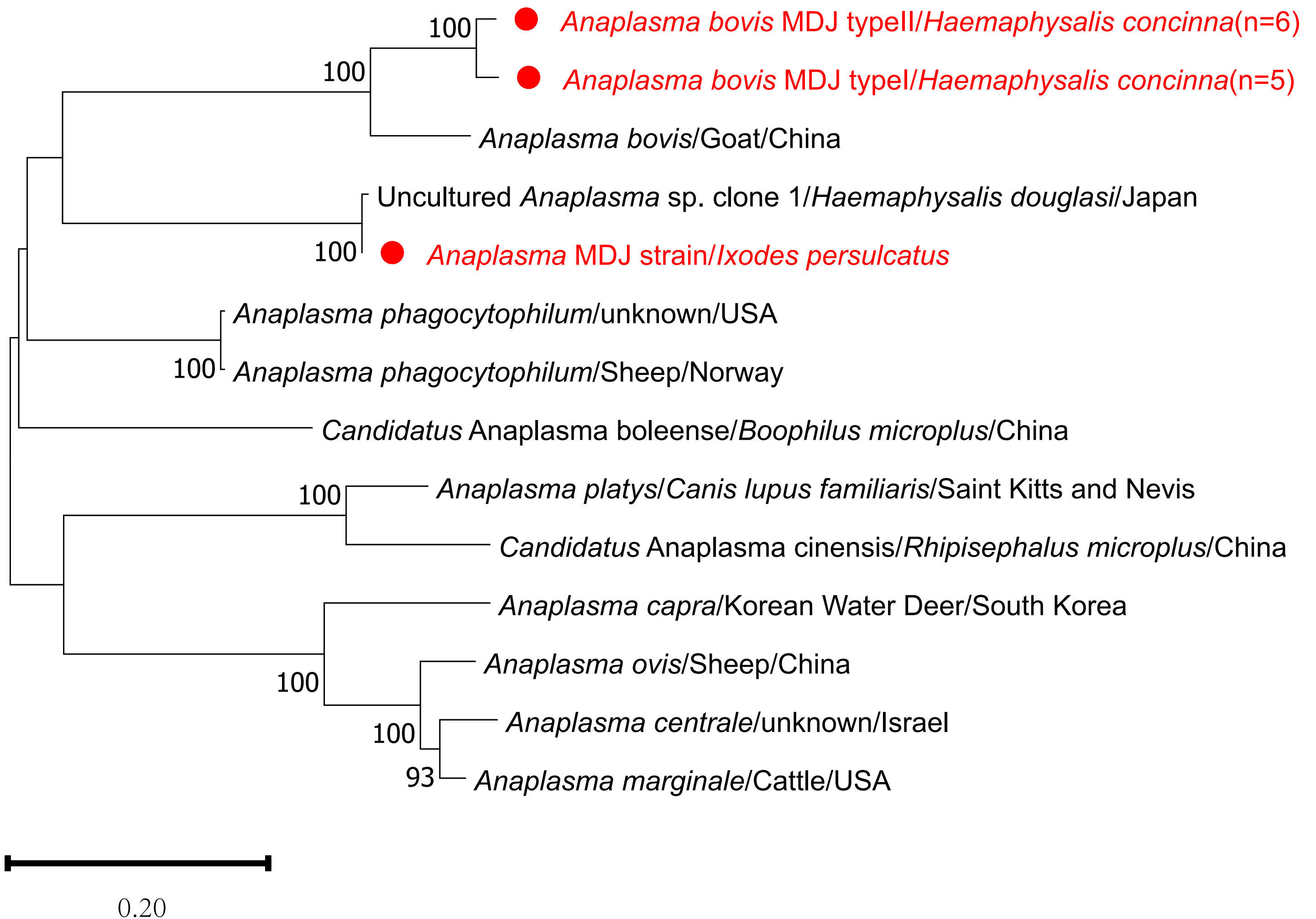

**Figure S1.** Phylogenetic tree based on concatenated nucleotide sequences of *Anaplasma*.

Supplement: Supplementary file 1 [file pathogens-15-00301-s001.zip › Figure S1.pdf]

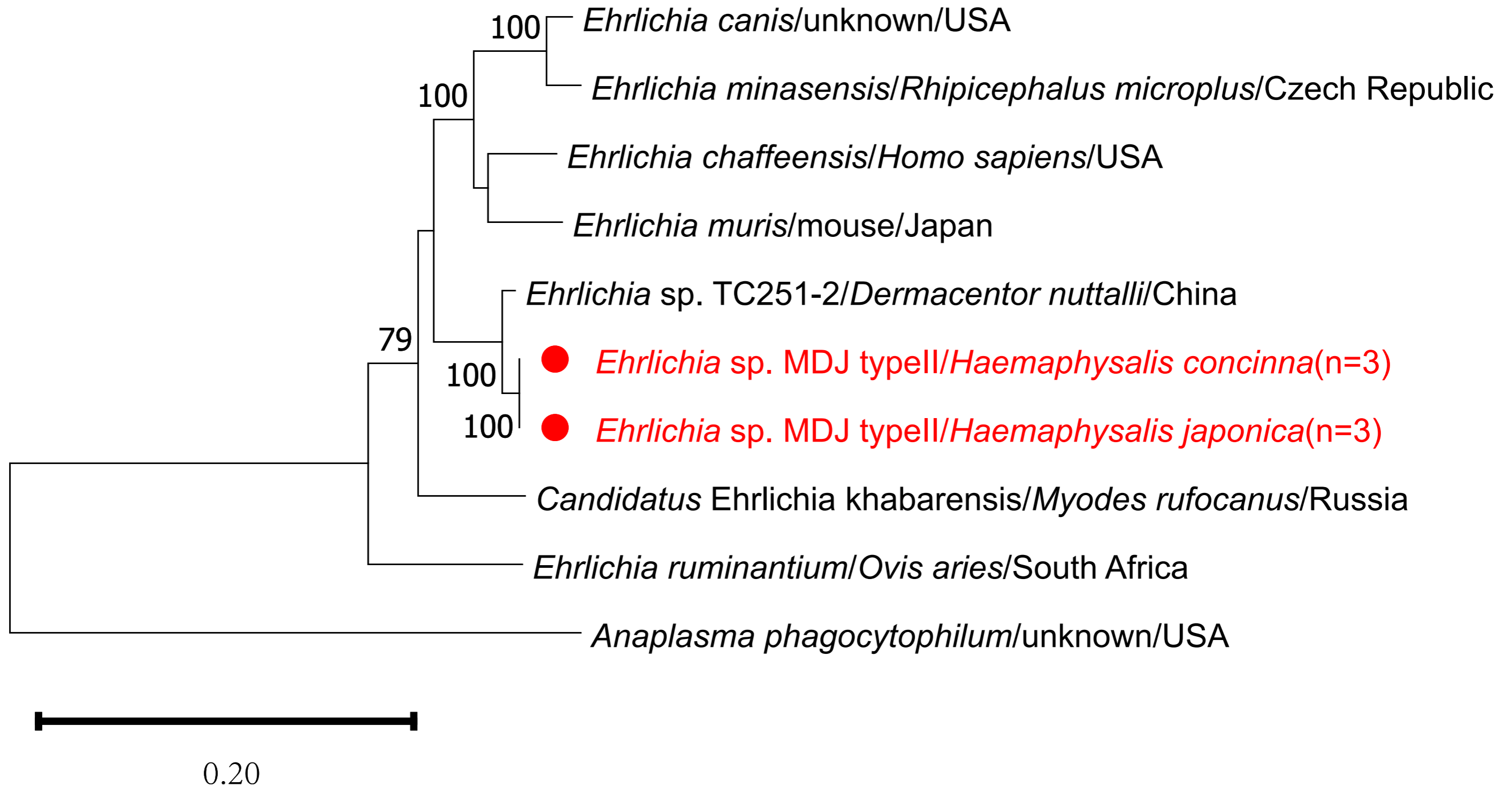

**Figure S2.** Phylogenetic tree based on concatenated nucleotide sequences of *Ehrlichia*.

Supplement: Supplementary file 1 [file pathogens-15-00301-s001.zip › Figure S2.pdf]
